# Predictive Model for the Surface Tension Changes of Chemical Solutions Used in a Clean-in-Place System

**DOI:** 10.3390/ma14133479

**Published:** 2021-06-22

**Authors:** Joanna Piepiórka-Stepuk, Monika Sterczyńska, Tomasz Kalak, Marek Jakubowski

**Affiliations:** 1 Department of Mechanical Engineering, Division of Food Industry Processes and Facilities, Koszalin University of Technology, Racławicka 15-17, 75-620 Koszalin, Poland; monika.sterczynska@tu.koszalin.pl (M.S.); marek.jakubowski@tu.koszalin.pl (M.J.); 2 Department of Industrial Products and Packaging Quality, Institute of Quality Science, Poznań University of Economics and Business, Niepodległości 10, 61-875 Poznań, Poland; tomasz.kalak@ue.poznan.pl

**Keywords:** cleaning in place system, cleaning agents, storage, surface tension, nonlinear regression

## Abstract

The paper presents the results concerning the influence of concentration and storage time on the equilibrium surface tension of chemical solutions used in a clean-in place (CIP) system. Standard cleaning solutions (prepared under laboratory conditions) and industrial solutions (used in a CIP system in a brewery) were subjected to tests. Solutions from the brewery were collected after being regenerated and changes in equilibrium surface tension were studied during a three-month storage. In the statistical analysis of the solutions, standard deviations were determined in relation to the averages, and a Tukey’s multiple comparison test was performed to determine the effect of dependent variables on the surface tension of solutions. From the results, a nonlinear regression model was developed that provided a mathematical description of the kinetics of changes in the wetting properties of the solutions during their storage. A linear–logarithmic function was adopted to describe the regeneration. Numerical calculations were performed based on the nonlinear least squares method using the Gauss–Newton algorithm. The adequacy of the regression models with respect to the empirical data was verified by the coefficient of determination R and the standard error of estimation Se. The results showed that as the concentration of the substance in the cleaning solution increased, its wetting properties decreased. The same effect was observed with increased storage time as the greatest changes occurred during the first eight weeks. The study also showed that the use of substances to stabilize the cleaning solutions prevented deterioration of their wetting properties, regardless of the concentration of the active substance or storage time.

## 1. Introduction

Chemical solutions are essential for cleaning processes irrespective of cleaning application (hand, foam or flow cleaning). The microbiological and physicochemical properties of cleaning substances determine their quality, usefulness and appropriateness for cleaning certain equipment and the post-production deposits that form on surfaces. These properties affect the cleanliness of surfaces, so solutions must first of all be free of microorganisms and solid deposits that could be a secondary source of surface contamination [1,2]. Additionally, they must meet a number of physicochemical requirements related to, among others, the appropriate concentration of active cleaning substances (alkaline, acidic or neutral) and the temperature at which they are used [3,4,5]. The values of these parameters are determined in the cleaning programs and mainly depend on the equipment being cleaned and the type of contaminants being removed: protein, fat or mineral [2]. The physicochemical properties of cleaning solutions (concentration, pH, conductivity, turbidity) are particularly important in a clean-in-place (CIP) system. This cleaning method is based on the flow of cleaning substances through the production equipment without the need to disassemble it. The values of the parameters, measured during cleaning, constitute the criteria for controlling this process [6,7]. Cleaning in the CIP system has found an application in many industries: chemical, petrochemical but mainly food. The advantage of this method is the possibility of the long-term storage (even up to 8 months) of ready-to-use cleaning solutions in the tanks of the CIP system and the possibility of their repeated use [8,9,10]. On the one hand, it reduces the costs associated with purchasing of chemicals and naturalizing wastewater, but on the other it obliges the user to regenerate solutions after each use and to monitor continuously the quality of concentration, conductivity, pH and microbiological purity [11,12,13,14,15].

**Figure 1:**
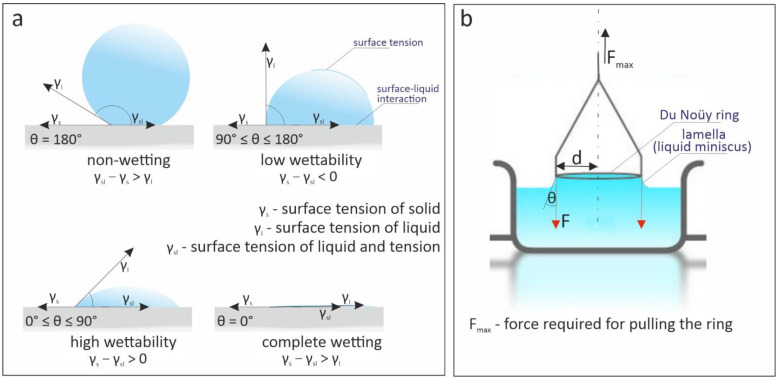
Surface–liquid interaction at the surface tension in (**a**) the contact angle method, and (**b**) the Du Noüy method.

One of the main factors affecting the CIP system is the ability of cleaning solutions to wet the deposits and surfaces. The wettability of a liquid droplet on a solid surface is determined by molecular interactions between structural materials and surfactants. These phenomena are governed by surface and interfacial interactions, typically acting at small (a few nanometers for van der Waals or electrostatic interactions) or very small (molecular) distances [16,17]. A strong attraction between liquid and solid molecules promotes the spreading of the liquid on the surface; conversely, a weak attraction causes the liquid droplet to flow. This property is often a criterion for optimizing chemical composition during the development of a new detergent and most often modeled in contact with the surface [18,19]. This property is also a criterion for predicting the behavior of washing solutions. Under static conditions, the wettability of liquids is commonly described in terms of the wetting angle θ (Figure 1a) by Young’s Equation (1), i.e., the lack of equilibrium cohesion forces between particles at the liquid–air interface. The components ϒ_s_, ϒ_sl_; and ϒ_l_ are the solid–air, solid–liquid, and liquid–air interfacial energies, respectively [20]. The wettability of the liquid can also be expressed by the surface tension (ƴ), also measured using static methods, among others, by measuring the force F_max_ needed to detach a platinum–iridium alloy ring of known radius *r* from a tested liquid surface (Figure 1b, Du Noüy’s method). In Equation (2) the value of the correction factor k = constant depends on the adopted calculation model and should be taken into account. The most common correction factors include those by Zuidema and Waters (used in measurements of liquids with low surface tension), Huh and Mason (which covers a wider range of liquids), and Harkins and Jordan.

(1) $$\cos\theta =\frac{\gamma_{s}-\gamma_{\mathrm{sl}}}{\gamma_{l}}$$

(2) $$\gamma =\frac{k\times F_{\max}}{4\times \pi \times d}$$

Cleaning solutions, owing to their surface activity, lower the solution–deposit–surface interfacial tension. They wet the organic and inorganic deposits formed in food production, facilitate deposit penetration, and penetrate all the folds and capillaries of the cleaned surfaces where water alone could not reach. As a result, deposit adhesion to the surface of production equipment is reduced; the deposits dissolve and move into the solution, where they are subject to emulsification, dispersion or micellar dissolution [6,21,22]. Studies conducted on changes to acidic and alkaline industrial solutions during use showed a decrease in the surface wetting angle ϴ and surface tension ƴ relative to standard solutions by about 10–50% [3,23]. In the case of soda lye, this phenomenon was explained by the hydrolysis of fat and proteins in post-production deposits (solutions collected from dairy plants), which produced surfactant molecules in the presence of NaOH (saponification). On the other hand, the lowering of the acidic solution’s surface tension was explained by an inadequate inter-operational rinsing between alkaline and acidic cleaning, which resulted in the transition of residual NaOH to H_3_PO_4_ + HNO_3_ solution. In addition, studies of agents collected from a brewery showed that these solutions exhibited reduced cleaning ability compared to their initial solutions [24]. The question, therefore, is whether changes in the surface tension of cleaning solutions occur solely as a result of reacting with deposits eluted from production installations or are also a result of the so-called aging of solutions during storage. There are no scientific reports in the literature on the frequency of agent replacement or the reasons that determine it. Replacement of cleaning agents in CIP stations is usually carried out at arbitrary periods (e.g., once every six months) or when microbiological contamination of the solution occurs. This research will extend knowledge on changes in wetting properties of cleaning solutions during their storage and regeneration. The work is experimental and is original in the light of ongoing research in the field of CIP cleaning. The results of the study come partly from a research grant related to the optimization of storage time of cleaning solutions in CIP station tanks.

## 2. Materials and Methods

### 2.1. Experimental Setup

This study included an experimental part and a statistical analysis of empirical data. The experimental study consisted of determining the changes in selected parameters of the cleaning solutions that occurred during storage. The statistical analysis included determining the nature of the changes to their wetting properties during storage. These were calculated as a function of their concentration on the basis of the non-linear regression function ƴ = f(t, C_p_), which is a predictive model for the kinetics of change in the equilibrium surface tension of solutions.

Concentrated acidic and alkaline cleaning solutions (strong acidic CIP cleaner based on HNO_3_—CLEAN A-N 30, ANTI-GERM International GmbH, Memmingen, Germany; strong alkaline CIP cleaner based on NaOH—Solchem Sp. z o.o., Bydgoszcz, Poland) used in a local brewery were evaluated. The alkaline solution was tested with and without the addition of a stabilizer according to industrial practice. The stabilizer (P3-stabicip OXI, Ecolab Europe, Wallisellen, Switzerland) was based on active oxygen (sodium peroxycarbonate) and contained surfactants and antifoam tensides (Ts) to increase wettability and prevent foaming during cleaning, respectively. All standard solutions were prepared from water collected from the brewery, which was also evaluated. The changes observed in the tested standard solutions were related to the changes observed in the solutions used for cleaning brewery installations.

From the time they were prepared, the standard solutions were stored under room conditions without access to light for 12 weeks. At the same time, industrial solutions were collected every other week, stored in CIP station tanks, and used for cleaning installations in the brewhouse. The H_3_PO_4_ + HNO_3_ (T—tanks) and NaOH (T—tanks) solutions were used to clean brewing vessels and the wort path, while the NaOH (F—filter) solution was used to clean the filter press. At the start of the study, these industrial solutions had already been in use for approximately three months. They were collected before the cleaning of production installations had started, i.e., after their regeneration and concentration. Both standard solutions (prepared in the laboratory) and industrial solutions (collected from the plant) were evaluated for their concentration (%), pH (-), turbidity (NTU), conductance (mS/cm) and density (g/cm^3^). These tests were performed at the beginning and after 12 weeks. In turn, changes in the surface tension of the solutions (mN/m) were studied at two-week intervals. The initial values of these parameters are presented in Table 1.

### 2.2. Determination of Solution Concentration

Solution concentration was performed by alkacimetric titration in the presence of phenolphthalein (Chempur, Piekary Śląskie, Poland). It consisted of neutralizing the cleaning solutions using appropriate titrants (pure p.a. HCl and NaOH, Chempur, Piekary Śląskie, Poland). The reactions and calculation of % concentration of individual solutions were performed according to the method presented by Piepiórka-Stepuk et al. [24]. The analyses of solutions at the initial and final storage periods were performed in triplicate. The results were averaged by determining the random error expressed as the standard deviation.

### 2.3. Measurement of pH and Conductivity

The conductivity of the solutions K (mS/cm) and pH were determined on a ProLab 2500 multimeter (SI Analytics, Mainz, Germany) by immersing the appropriate electrodes and temperature sensor in the test samples. Changes in temperature were automatically converted into the values of the parameters tested (conductance, pH). The tests for each solution in the initial and final storage periods were performed in triplicate, and the results were statistically analyzed by determining the random error and standard deviation.

### 2.4. Measurement of Solutions Turbidity

Turbidity measurements were performed with a Lovibond TB 300 IR turbidity meter (Tintometer Inc., Dortmund, Germany) in scattered light with a wavelength of λ = 860 nm (infrared light) at an angle of 90°. The principle of the method, according to ISO norm [25] is based on the scattering and absorption of radiation as the light beam passes through the dispersion system. The intensity of the scattered light increases with turbidity. For this purpose, a cuvette with the test solution was placed in a measuring cell and subjected to the analysis. Before the beginning of each test cycle, the sensor was calibrated on Formazine (Tintometer Inc., Dortmund, Germany) in the range of 0–1100 NTUs (Nephelometric Turbidity Units). The testing of solutions at the beginning and end of the storage was performed in triplicate. The results were averaged by determining the random error expressed as the standard deviation.

### 2.5. Measurement of Solutions Density and Their Equilibrium Surface Tension

The measurement was performed in a water/air system by the du Noüy method on an automatic D—MT1A tensiometer (Donserv, Warsaw, Poland) with a Peltier system. The method measures the force required to detach a platinum ring from a liquid surface. For this purpose, the ring was immersed in a sample of a tested solution and then raised at a speed of 0.1 mm/s to a height that ensured an equilibrium state, where the force required to break the micelle formed between the surface of the ring and the test liquid was at its maximum value. At this point, the equilibrium surface tension ƴ (mN/m) was read. The measurements were performed at room temperature 20 ± 2 °C. The tests of each solution were performed in six repetitions every other week over a period of three months (0, 2, 4, 6, 8, 10 and 12 weeks), and the results were analyzed further.

### 2.6. Statistical Analysis

Statistical analyses were conducted to determine the nature of the effects of individual variables on changes in equilibrium surface tension and their mutual interaction.

#### 2.6.1. Determination of the Nature of the Interactions of Solution Concentration and Storage Time on Their Wetting Properties

The results were averaged and presented on graphs as kinetics of change in equilibrium surface tension during the 12 weeks of storage and as a function of concentration, and standard deviations were determined. To test the significance of the effect of storage time on the equilibrium surface tension, a Fisher–Snedecor test was performed in Statistica 13.1 software (2016, StatSoft, Kraków, Poland) to test the null H_0_: (μ = μ_0_) and alternative hypothesis H_1_: (μ ≠ μ_0_), with F_kryt_ calculated for each solution and presented in the results section. A Tukey’s multiple comparisons test (post hoc HSD—honest significant difference test) was also performed at a significance level of α = 0.05. From the scatter curves, the nature of the effect of each independent variable on the changes in surface tension ƴ = f(t); ƴ = f(C_p_) was determined.

#### 2.6.2. Development of Predictive Models for Particular Solutions ƴ = f(t, C_p_)

The modeling of the function ƴ = f(t, C_p_) for each of the solutions and its fitting were performed by a nonlinear least squares estimation using the Gauss–Newton algorithm [9]. The procedure for matching the analytical formula to the empirically determined functional relationship consisted of two major stages. In the first stage, the form of the formula was selected; in the next stage, the numerical values of the relevant parameters (coefficients) that approximated the objective function to the expected course the closest were estimated [26]. The adequacy of the adopted model was checked by the goodness of fit of the predicted values with respect to the observed ones by determining the coefficient of determination R and the standard error of estimation Se. The results of these calculations give the response surfaces between the independent variables and the dependent variable determined for the three tested cleaning solutions.

## 3. Results and Discussion

### 3.1. Changes Observed in Solutions after Their Storage Time

**Figure 2:**
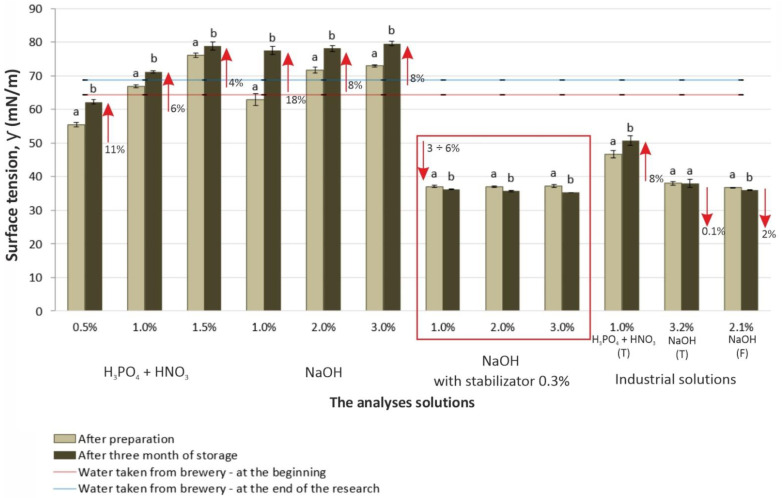
Changes in surface tension of the tested solutions during their storage (^a, b^ different letters in the same column indicate significant statistical difference Tukey’s HSD test, α = 0.05).

The average values of the equilibrium surface tension of the solutions of fixed concentrations and the changes in this parameter during the storage are presented in Figure 2.

In the case of both acidic and alkaline solutions without a stabilizer, the value of their surface tension ƴ rose with increasing C_p_ concentration: for the acidic solutions it was 55.5–76.0 mN/m; for alkaline ones, 62.9–72.9 mN/m. The dependence of C_p_ on ƴ is related to the phenomenon of solute adsorption at the interface and depends on the concentration of this substance in the surface layer being higher or lower than within the solution. This in turn depends on the interaction of water molecules with the solute molecules. It could be assumed that the ions of the tested substances were hydrophilic (had a strong affinity for polar water molecules) and were drawn deep into the solution; therefore, the strong electrolyte solutions had a higher surface-tension value than that of water. Moreover, the surface tension ƴ for the same solutions increased significantly after 12 weeks of storage: 4–11% for acidic and 8–18% for alkaline solutions, respectively (Tukey’s HSD post hoc test results). Concurrently, the lower C_p_ of the solution the greater the increase in ƴ, and thus the lower the ability of the solution to wet the surface.

Interesting results were obtained for alkaline solutions with NaOH + S stabilizer. Irrespective of their concentration C_p_, the value of surface tension ƴ of these solutions remained within 37.02–37.20 mN/m and was much lower than that of the alkaline solutions without stabilizer. The tensides contained in this solution included both polar hydrophilic groups with an affinity for water, and nonpolar—lipophilic (hydrophobic) groups, which repel water. The liking of the hydrophobic part of the tenside directly to the functional group of the cleaning solution created negative ions, which is what finally reduced the surface tension. In addition, during storage this value still decreased in a statistically significant manner (3–6%) in relation to the initial solutions, which is beneficial from the point of view of their cleaning properties. A similar phenomenon was observed in the case of industrial alkaline solutions.

The results for the industrial solutions were further analyzed in relation to the standard solutions. It should be emphasized that at the beginning of the study the industrial solutions had already been used in the plant for three months and had been prepared from the same commercial preparations using the same solvent (water) as the standard solutions. In addition, the NaOH (T) and NaOH (F) solutions were enriched with a stabilizing preparation at the same concentration as the standard solutions––0.3%. The industrial alkaline solutions did not differ significantly in surface tension from their standard solutions with stabilizer (C_p_ 2 and 3%). Like these, these had a reduced ƴ value of about 0.1–2.0% after 12 consecutive weeks of use. This was consistent with previous reports by the authors [24]. In the literature, this phenomenon is explained by the hydrolysis of fat and proteins in the post-production deposits, which in the presence of NaOH produced surfactant molecules (soap), which lowered the surface tension [3,21,23,27]. However, with respect to the results, it seems that this was not the only cause: the standard solutions were not used in the cleaning process, and yet their surface tension also decreased (by about 3–6%).

The industrial acidic solution H_3_PO_4_ + HNO_3_ (T) had a different surface tension (about 30% lower) compared to the 1% standard solution H_3_PO_4_ + HNO_3_. This result was consistent with previous reports [24,28], and may have been a consequence of inadequate inter-operative rinsing between alkaline and acidic cleaning, resulting in the passage of residual NaOH solution into the H_3_PO_4_ + HNO_3_ solution. After 12 weeks of storage, this solution showed an approximate 8% increase in surface tension values, which is consistent with the results for its reference counterpart.

The study of other parameters characterizing the tested solutions after the 12 weeks were not statistically significant (Table 2).

Based on the above analysis, it can be assumed that the surface tension changes in the standard solutions were adequate to the changes that occurred in the industrial solutions. This is of key importance for modeling and optimization of the aging of cleaning solutions, as it enables the development of a model based on the results for standard solutions.

### 3.2. Analysis of the Nature of Interactions of Independent Variables on the Wetting Properties of Solutions

**Figure 3:**
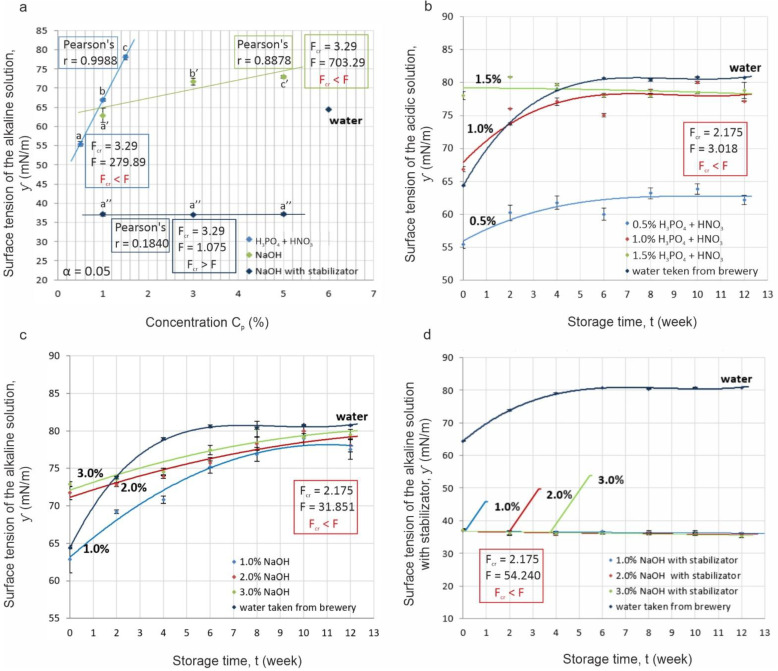
Relationships of equilibrium surface tension values with: (**a**) concentration of solutions (C_p_); (**b**) storage time (t) of acidic solution H_3_PO_4_ + HNO_3_; (**c**) storage time (t) of alkaline solution NaOH; (**d**) storage time (t) of alkaline solution with stabilizer NaOH + S (^a–c; a′–c′; a″–c″^ different letters in the same column indicate significant statistical difference Tukey’s HSD test, α = 0.05).

Figure 3 shows the results of the statistical analyses and the dependence of surface tension on the concentration and storage time of the solutions.

It was observed that the surface tension of acidic and alkaline solutions without stabilizer rose with the increases in their working concentration. The results of the Fisher’s test for the experimental results (for significance level α = 0.05), allowed for the rejection of the null hypothesis (H_0_: (μ = μ_0_)), the equality of surface tension for solutions of different concentrations stored over the 12 weeks, and accept the alternative hypothesis H_1_: (μ ≠ μ_0_). For each solution analyzed, the values of the F-statistic (F_Concentration (α = 0.05; df1 = 3; df2 = 33)_ = 3.290; F_Time (α = 0.05; df1 = 6; df2 = 119)_ = 2.1750) satisfied the inequality (F_kryt_ < F) (Figure 3a–d). Thus, the results indicated a significant effect of the analyzed dependent variables (C_p_, t) on the wetting properties of the solutions, which was expressed as equilibrium surface tension (Figure 3a–c). Only when stabilizer is added to a solution should the null hypothesis be accepted because it would mean that the equilibrium surface tension of the solution was invariant and independent of concentration (Figure 3a). The absence of this relationship was also demonstrated by the value of the Person correlation coefficient (r = 0.1840) between the variables ƴ and C_p_. At the same time, this result confirmed the effectiveness and validity of using additives to stabilize cleaning solutions immediately after preparation. However, the lack of homogeneity in the solutions with the added stabilizer was demonstrated during =storage, which allowed the null hypothesis to be rejected (Figure 3d). The values of the Person correlation coefficients for these variables (ƴ, t) were negative and varied from r = −0.6572 to −0.8295, depending on C_p_, which indicated a strong negative correlation among the analyzed variables and should be interpreted as follows.

With storage time, the equilibrium surface tension of the stabilized alkaline solutions decreased, which is favorable for cleaning because the ability of the solutions to wet the surface is increased. For acidic and alkaline formulations without stabilizer, equilibrium surface tension increased along with the solution concentration. The Pearson correlation coefficient values for these variables (ƴ; C_p_) were obtained at r = 0.9988 and 0.8878, respectively. Similarly, strong correlations of surface tension changes in these solutions were shown as a function of storage time (ƴ, t). In this case, the values of the Person correlation coefficients for acidic solutions varied in the range r = 0.7285–0.7943, and for alkaline solutions r = 0.9264–0.9375, which meant that the wetting properties of these solutions deteriorated during storage.

On the basis of the above results, the nature of the interactions of the analyzed parameters on equilibrium surface tension was determined. The distribution of the results suggested that the effect of solution concentration on wettability, expressed as equilibrium surface tension ƴ = f(C_p_), could be approximated by a linear function (Figure 3a), while the effect of storage time ƴ = f(t) was shown by a logarithmic function (Figure 3b–d), which was in agreement with the results of other authors [24]. The distributions of the dependent variables were used as assumptions for the development of the final model, which described the relation ƴ = f(C_p_; t). On the basis of this, it was possible to determine the simultaneous effect of the dependent variables (t, C_p_) on the ability of solutions to wet the cleaned surfaces, expressed as the equilibrium surface tension.

### 3.3. Analysis of the Final Predictive Model ƴ = f(C_p_, t)

Based on the above analyses, three predictive, nonlinear regression models (ƴ = f(C_p_, t)) were developed that mathematically described the changes in the equilibrium surface tension of the cleaning solutions during their storage in the CIP station tanks. A mathematical model of dynamic objects for nonlinear regression based on the nonlinear east squares (NLS) method was adopted to describe the phenomenon. The idea of the NLS method is to minimize the sum of the squares of differences between the actual and theoretical values of the explanatory variable. The method approximates the function Y(x_i_) = y_i_ over a discrete set x_i_ in an interval of variables (in this case, t and C_p_) to perform iterative estimated calculations based on a linearized nonlinear model. A linear–logarithmic function with single interactions (Equation (3)) was chosen for the description, with the coefficients a, b, c, assumed to account for the interactions of the analyzed parameters on the final effect of changes. The equation has three basic factors (a, b and c), which determine the shape of the function such that its course corresponds to changes in quantity over time t. The coefficient “a” determines the initial value for the surface tension of a specific cleaning solution; ”b” is the influence of time for the logarithmic function; and “c” is the influence of the concentration of a cleaning solution for the linear function, but it can be treated as the “weight coefficient” because of the linear effect of the cleaning solution concentration on the surface tension. The coefficient values were determined from statistically collected numerical data by the NLS method using the Gauss–Newton algorithm. The regression functions have both theoretical and practical significance.

ƴ = f(C_p_, t) = a + b × log(t) + c × C_p_ (3)

This form of the function means that at time t = 0, the equilibrium surface tension depends only on the concentration of the solution. The functions represent changes in surface tension (ƴ) of acidic (H_3_PO_4_ + HNO_3_) and alkaline (NaOH; NaOH + S) solutions as a function of their concentration and storage time ƴ = f(t, C_p_). Finally, the functions take the following form (Equations (4)–(6)):

ƴ_H3PO4 + HNO3_ = f(t; C_p_) = 53.47 + 0.89 × log(t) + 0.24 × C_p_   R = 0.852; Se = 0.726 (4)

ƴ_NaOH_ = f(t; C_p_) = 69.47 + 1.98 × log(t) + 1.98 × C_p_         R = 0.758; Se = 0.575 (5)

ƴ_NaOH + S_ = f(t; C_p_) = 36.84 − 0.22 × log(t) − 0.15 × C_p_       R = 0.783; Se = 0.613 (6)

**Figure 4:**
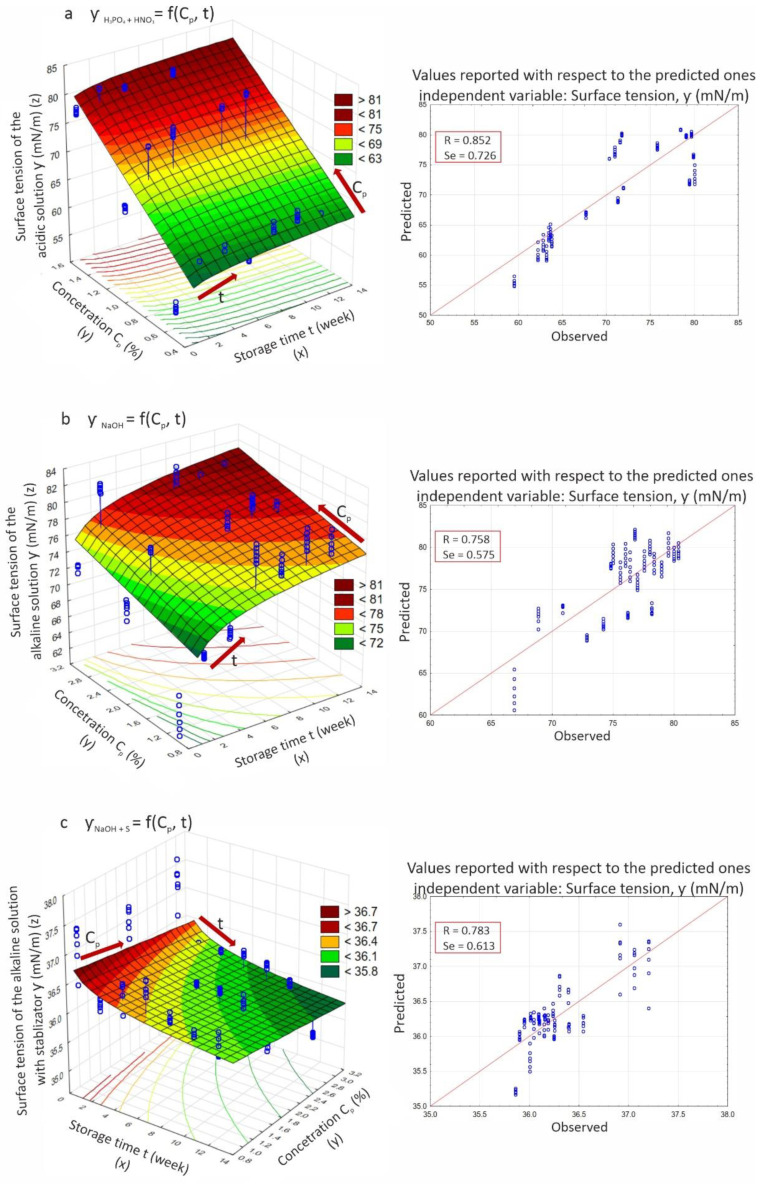
Relationship between equilibrium surface tension and storage time of solutions of different concentrations: (**a**) acidic, based on a 1:1 mixture of H_3_PO_4_ + HNO_3_; (**b**) alkaline, based on NaOH; (**c**) alkaline, based on NaOH with stabilizer.

These functions are graphically presented in Figure 4a–c as response plots of the predicted equilibrium surface tension values (ƴ) for the solutions, with different concentrations (C_p_) relative to the value observed during their storage time (t).

The goodness of fit of the adopted function model was also presented on the basis of the spread between predicted and observed values. Validation of the regression model was based on test results for the industrial solutions. The results are presented in Table 3, where equilibrium surface tension data that differs significantly from the predicted model values are highlighted in red. On their basis, it can be concluded that the industrial cleaning solutions (repeatedly regenerated and reused over six months) did not differ significantly in equilibrium surface tension from their standard counterparts. Significant differences were found only for three samples of the acidic solution. Thus, the developed models for standard solutions can be used to predict changes in industrial solutions. The coefficient values of fit R of the regression models (Equations (3)–(5)) to the empirical data are high and in the range R = 0.758–0.852, while the standard deviation values of the residuals, called standard error of estimation (Se), were in the range Se = 0.575–0.726, which also indicates a fairly good fit of the models to the empirical data.

The response surfaces determined the nature of the interactions of the individual factors on the wetting properties of the solutions. They showed that as the concentration of acidic and alkaline cleaning solutions increased so did their equilibrium surface tension; thus, their wetting properties decreased. This was consistent with many reports [5,23,27,28,29,30,31]. This effect was intensified by the storage time, which was particularly noticeable in the first weeks. In contrast, the addition of a stabilizing substance completely reversed the effects of time and solution concentration on equilibrium surface tension. In this case, both the increase in concentration and storage time influenced the decrease in surface tension values. The effect of these interactions is indicated by an arrow in Figure 4.

## 4. Conclusions

The cleaning solutions used in the CIP system of production installations differed in their wetting properties as expressed by equilibrium surface tension. This depended on the type of active substance and its concentration in the working solution, but also on the amount of time for which the solution was stored. Taking into account equal concentration of the active substance, one can say that acidic solutions are characterized by a higher surface tension than alkaline ones, which proves their weaker wettability. Moreover, it was found that the higher the concentration of active substance in a solution (alkaline or acidic) the higher the surface tension. A significant role in this respect is played by the stabilizing substances, the addition of which (in the case of one based on active oxygen) almost caused a two-fold decrease in the surface tension of an alkaline solution and stabilized it throughout the study period. The study also demonstrated that the storage time also influenced changes in wetting properties. In this respect it was shown that after about 4–8 weeks, there was a statistically significant increase in the equilibrium surface tension of solutions without a stabilizer compared to their initial solutions. At the same time, the dynamics of these changes depended on their working concentration—the lower the concentration, the faster the changes. Hence, to maintain or restore the wetting properties of solutions, it was necessary to use additional substances, the so-called stabilizers.

The study of the industrial solutions also showed that repeated use with this method caused a slight deterioration in equilibrium surface tension. Significant deterioration was shown only for the acidic solution. Taking into account the authors’ earlier studies [3,23,28] concerning the presence of colloidal suspensions in solutions (despite their regeneration and the difficulties in maintaining the proper microbiology), solutions aimed at extending their useful life should be more widely sought. The nature of these changes is described in the authors’ previous works [8]. Furthermore, from the results of both the wetting properties of solutions without a stabilizer and their purity, it can be assumed that the long-term use of such solutions for cleaning in CIP poses a great threat to food production hygiene. This threat comes from the possibility of insufficient cleaning of production equipment surfaces and secondary contamination by solid deposits.

The nature of changes to the equilibrium surface tension of cleaning solutions during their storage in CIP station tanks, depended on their concentration, which was described by the linear–logarithmic equation. The relationship was characterized by a good fit to empirical data. The values of determination indices for the equations were high and could be treated as an adequate mathematical model that described the changes in equilibrium surface tension of chemical cleaning solutions during their storage. Further research should focus on the inclusion of temperature interactions in the equations. Ultimately, the equations provided a criterion for optimizing the storage time of the solutions in the CIP station tanks.

## Figures and Tables

**Table 1:** Properties of CIP solutions.

Type of Solution		Concentration (*v*/*v*) (%)	pH (-)	Conductivity (mS/cm)	Turbidity (NTU)	Surface Tension (mN/m)	Density (g/cm^3^)
Standard	1. Acidic 1:1 (H_3_PO_4_ + HNO_3_)	0.50 ± 0.01	1.63 ± 0.00	12.68 ± 0.00	0.25 ± 0.02	55.45 ± 0.63	0.98 ± 0.02
		1.00 ± 0.01	1.41 ± 0.01	25.34 ± 0.02	0.24 ± 0.01	66.85 ± 0.38	1.01 ± 0.00
		1.51 ± 0.02	1.23 ± 0.00	37.99 ± 0.01	0.24 ± 0.02	76.03 ± 0.63	1.01 ± 0.01
	2. Alkaline (NaOH)	1.00 ± 0.01	12.91 ± 0.00	45.33 ± 0.02	11.37 ± 0.25	62.89 ± 1.82	0.99 ± 0.01
		2.00 ± 0.01	12.97 ± 0.01	81.76 ± 0.01	12.17 ± 0.05	71.74 ± 0.90	1.02 ± 0.00
		3.02 ± 0.01	12.99 ± 0.00	120.07 ± 0.02	24.37 ± 0.09	72.89 ± 0.35	1.03 ± 0.00
	3. Alkaline (NaOH with stabilizator 0.3%)	1.01 ± 0.01	12.76 ± 0.03	42.15 ± 0.00	14.63 ± 0.12	37.06 ± 0.37	1.01 ± 0.01
		2.01 ± 0.01	12.83 ± 0.01	80.89 ± 0.01	66.37 ± 0.33	37.02 ± 0.21	1.02 ± 0.00
		3.02 ± 0.01	12.82 ± 0.02	114.47 ± 0.02	96.47 ± 0.48	37.19 ± 0.34	1.04 ± 0.01
Industrial	4. Phosphoric and nitric acid 1:1 H_3_PO_4_ + HNO_3_ (T)	1.02 ± 0.01	1.34 ± 0.01	26.61 ± 0.01	4.13 ± 0.06	46.57 ± 1.11	1.01 ± 0.01
	5. Sodium hydroxide—NaOH (T)	3.21 ± 0.01	12.86 ± 0.01	115.41 ± 0.04	1.25 ± 0.02	37.95 ± 0.59	1.04 ± 0.01
	6. Sodium hydroxide—NaOH (F)	2.08 ± 0.01	12.93 ± 0.01	85.21 ± 0.04	16.50 ± 0.02	36.67 ± 0.05	1.03 ± 0.01
	Water	-	7.55 ± 0.01	0.54 ± 0.00	0.11 ± 0.01	64.39 ± 0.11	0.99 ± 0.01

**Table 2:** Properties of CIP solutions after 12 weeks of storage.

Type of Solution		Concentration (*v*/*v*) (%)	pH (-)	Conductivity (mS/cm)	Turbidity (NTU)	Surface Tension (mN/m)	Gęstość (g/cm^3^)
Standard solutions	1. Acidic 1:1 (H_3_PO_4_ + HNO_3_)	0.50 ± 0.01	1.78 ± 0.00	12.59 ± 0.00	0.18 ± 0.01	62.20 ± 0.72	1.01 ± 0.00
		1.00 ± 0.01	1.56 ± 0.01	25.24 ± 0.01	0.32 ± 0.01	71.19 ± 0.35	1.01 ± 0.00
		1.51 ± 0.02	1.29 ± 0.00	37.29 ± 0.85	0.22 ± 0.01	78.80 ± 1.19	1.01 ± 0.00
	2. Alkaline (NaOH)	1.00 ± 0.01	12.87 ± 0.03	44.24 ± 0.07	13.44 ± 0.36	77.53 ± 1.29	1.02 ± 0.01
		2.00 ± 0.01	12.98 ± 0.01	83.20 ± 0.03	13.61 ± 0.22	78.07 ± 0.89	1.03 ± 0.01
		3.02 ± 0.01	13.01 ± 0.01	117.81 ± 0.07	25.48 ± 0.78	79.51 ± 0.69	1.04 ± 0.01
	3. Alkaline (NaOH with stabilizator 0.3%)	1.01 ± 0.01	12.97 ± 0.03	42.25 ± 0.12	65.48 ± 2.69	36.16 ± 0.16	1.02 ± 0.00
		2.01 ± 0.01	12.95 ± 0.01	80.60 ± 0.01	64.01 ± 0.38	35.68 ± 0.14	1.03 ± 0.00
		3.02 ± 0.01	12.98 ± 0.02	113.51 ± 0.04	61.54 ± 0.78	35.21 ± 0.03	1.03 ± 0.01
Industrial solutions	4. Phosphoric and nitric acid 1:1 H_3_PO_4_ + HNO_3_ (T)	1.12 ± 0.01	1.66 ± 0.03	26.32 ± 0.04	2.23 ± 0.14	50.67 ± 1.42	1.01 ± 0.01
	5. Sodium hydroxide—NaOH (T)	3.20 ± 0.01	13.06 ± 0.03	112.54 ± 1.40	1.39 ± 0.06	37.92 ± 1.18	1.05 ± 0.00
	6. Sodium hydroxide—NaOH (F)	2.12 ± 0.01	13.05 ± 0.01	82.81 ± 0.03	17.34 ± 0.10	35.94 ± 0.15	1.03 ± 0.00
	Water	-	7.32 ± 0.02	0.52 ± 0.00	0.15 ± 0.02	68.67 ± 0.17	1.02 ± 0.01

**Table 3:** Significant differences between empirical data and model results (Tukey’s HSD test, α = 0.05).

	Type of Solution	Time of Storage (Week)						
		0	2	4	6	8	10	12
**Surface tension (ƴ)** **(mN/m)**	Industrial 1% H_3_PO_4_ + HNO_3_	46.57 ± 1.11	49.70 ± 1.25	46.24 ± 0.70	48.88 ± 1.58	48.40 ± 0.84	51.39 ± 1.42	50.67 ± 1.42
	Model 1% H_3_PO_4_ + HNO_3_	52.82 ± 3.91	53.98 ± 3.99	54.25 ± 4.01	54.40 ± 4.03	54.51 ± 4.03	54.60 ± 4.04	54.67 ± 4.05
	Industrial 3.2% NaOH + S	37.75 ± 0.59	39.64 ± 1.03	39.10 ± 0.87	39.89 ± 0.59	38.90 ± 0.98	38.75 ± 0.87	38.92 ± 1.18
	Model 3.2% NaOH + S	36.58 ± 3.97	36.29 ± 3.94	36.23 ± 3.93	36.19 ± 3.93	36.16 ± 3.92	36.14 ± 3.92	36.12 ± 3.92
	Industrial 2.1% NaOH + S	36.67 ± 0.05	36.65 ± 0.14	36.83 ± 0.10	36.65 ± 0.08	36.23 ± 0.07	37.35 ± 1.24	35.94 ± 0.15
	Model 2.1% NaOH + S	36.75 ± 3.99	36.46 ± 3.96	36.39 ± 3.95	36.35 ± 3.94	36.33 ± 3.94	36.31 ± 3.94	36.29 ± 3.94

## Data Availability

Data is contained within the article.
